# Differential Occurrence of Interactions and Interaction Domains in Proteins Containing Homopolymeric Amino Acid Repeats

**DOI:** 10.3389/fgene.2015.00345

**Published:** 2015-12-18

**Authors:** Ilaria Pelassa, Ferdinando Fiumara

**Affiliations:** ^1^Department of Neuroscience, University of TorinoTorino, Italy; ^2^National Institute of Neuroscience (INN)Torino, Italy

**Keywords:** amino acid repeats, homopolymeric, polyglutamine, polyalanine, protein-protein interactions

## Abstract

Homopolymeric amino acids repeats (AARs), which are widespread in proteomes, have often been viewed simply as spacers between protein domains, or even as “junk” sequences with no obvious function but with a potential to cause harm upon expansion as in genetic diseases associated with polyglutamine or polyalanine expansions, including Huntington disease and cleidocranial dysplasia. A growing body of evidence indicates however that at least some AARs can form organized, functional protein structures, and can regulate protein function. In particular, certain AARs can mediate protein-protein interactions, either through homotypic AAR-AAR contacts or through heterotypic contacts with other protein domains. It is still unclear however, whether AARs may have a generalized, proteome-wide role in shaping protein-protein interaction networks. Therefore, we have undertaken here a bioinformatics screening of the human proteome and interactome in search of quantitative evidence of such a role. We first identified the sets of proteins that contain repeats of any one of the 20 amino acids, as well as control sets of proteins chosen at random in the proteome. We then analyzed the connectivity between the proteins of the AAR-containing protein sets and we compared it with that observed in the corresponding control networks. We find evidence for different degrees of connectivity in the different AAR-containing protein networks. Indeed, networks of proteins containing polyglutamine, polyglutamate, polyproline, and other AARs show significantly increased levels of connectivity, whereas networks containing polyleucine and other hydrophobic repeats show lower degrees of connectivity. Furthermore, we observed that numerous protein-protein, -nucleic acid, and -lipid interaction domains are significantly enriched in specific AAR protein groups. These findings support the notion of a generalized, combinatorial role of AARs, together with conventional protein interaction domains, in shaping the interaction networks of the human proteome, and define proteome-wide knowledge that may guide the informed biological exploration of the role of AARs in protein interactions.

## Introduction

Homopolymeric amino acid repeats (AARs) are found in a large number of eukaryotic proteins (Faux, 2012). These repetitions in the primary sequence of proteins have been initially understood simply as unstructured “spacers” between protein domains or even just as “junk” peptides devoid of specific functions (Green and Wang, 1994; Karlin and Burge, 1996; as discussed in Haerty and Golding, 2010), but prone in some cases to misfolding, as in genetic diseases related to the expansion of polyglutamine (polyQ) or polyalanine (polyA) repeats (Almeida et al., 2013). A growing body of evidence is changing these views by showing how at least some of these repeats have defined structural propensities and functional properties. For instance, we have recently found that polyQ and polyA repeats can form coiled coil supersecondary structures which can regulate the oligomerization, interactions, and functions of proteins (Fiumara et al., 2010; Pelassa et al., 2014). Several studies have now explored the functional consequences of the appearance and variation in length of AARs in transcription factors and in other proteins in which they are particularly enriched, showing how these repeats can alter the function of proteins, thus ultimately modulating developmental and post-developmental processes (e.g., Fondon and Garner, 2004; Anan et al., 2007; O'Malley and Banks, 2008; Nasu et al., 2014).

One of the possible mechanisms by which AARs could regulate the function of proteins that contain them is by mediating the interactions of these proteins with other proteins or with other cellular components such as nucleic acids and lipids in membranes. In support of this hypothesis, we have shown for example that polyQ or polyA repeats can mediate interactions between proteins that contain them (e.g., Fiumara et al., 2010; Pelassa et al., 2014), while polyproline (polyP)-II structures and proline-rich sequences can mediate protein-protein interactions by binding to non-repetitive interaction domains (Yu et al., 1994). Evidence exists that some charged AARs may also drive protein-nucleic acid and protein-lipid interactions (Dean, 1983; Nam et al., 2001; DeRouchey et al., 2013).

AARs and conventional protein-protein, -nucleic acid, and -lipid interaction domains, are often found together in the same proteins. Thus, AARs and non-repetitive, conventional interaction domains may work combinatorially in defining the overall specificity and strength of the interactions of their parent proteins with other proteins or with other interaction partners. Initial evidence indicates indeed the possibility that AARs in proteomes, also together with non-repetitive sequences, may participate in the definition of entire protein-protein interaction networks. For example, it has been shown that disease-related and other polyQ proteins could drive the formation of protein-protein interaction networks based on coiled coil-mediated interactions (Fiumara et al., 2010; Petrakis et al., 2012; Schaefer et al., 2012) and this may also be the case for polyA proteins (Pelassa et al., 2014).

It is still unclear, however, to what degree the emerging roles of polyQ AARs in shaping protein-protein interaction networks in proteomes may be generalized to other AARs. Can other AARs also drive the formation of protein-protein interaction networks? And, with which conventional protein interaction domains may AARs cooperate in establishing these interactomes? The answer to these questions must ultimately come from biological experiments. However, given the scale and complexity of the biological problems raised by such questions, proteome-wide bioinformatics screenings may be essential for guiding the informed biological exploration of all the possible roles of AARs in establishing protein-protein interaction networks, also together with conventional protein interaction domains.

Based on these premises, we have undertaken here a quantitative bioinformatics analysis of the protein-protein interaction networks formed by the proteins containing AARs of each one of the 20 amino acids. Furthermore, we have determined whether specific protein-protein, -nucleic acid, and -lipid interaction domains are overrepresented in each one of the 20 AAR-containing protein groups. The results of our analyses overall provide quantitative support to the hypothesis that, together with conventional protein interaction domains, AARs may play a generalized, combinatorial role in establishing protein-protein interaction networks.

## Results

### Analysis of interactomes reveals differential connectivity in AAR-containing protein groups

**Figure 1:**
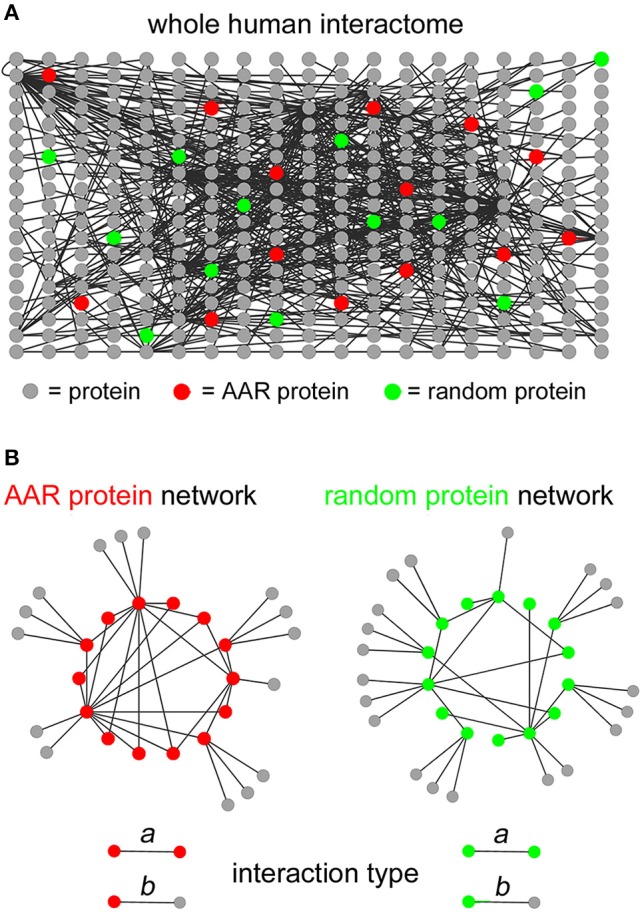
**Extraction of interaction networks of AAR proteins from the total human interactome. (A)** Schematic simplified representation of the human interactome in graph form. *Gray circles* represent proteins and *black lines* represent binary interactions between proteins as derived from the BioGrid database. *Red circles* represent proteins containing a given AAR. *Green circles* represent proteins selected randomly as a control group for the AAR protein group. **(B)** Simplified schemes representing in graph form (*left scheme*) the interaction network formed by proteins containing a given AAR (*red circles*) and their interactors (*gray circles*), or (*right scheme*) the interaction network formed by randomly selected proteins (*green circles*) and their interactors (*gray circles*). The lower part of the panel shows the two types of interactions that were defined in the interactomes above. Type *a* interactions occur between two AAR-containing proteins or between two proteins of the randomly selected control group. Type *b* interactions occur between an AAR-containing protein and an interactor that does not contain the repeat, or between a protein of the random control group and an interactor that is not part of the random protein group.

To determine the potential involvement of AARs in establishing protein-protein interaction networks, we first analyzed the interactomes formed by the proteins of each of the 20 groups of proteins of the human proteome containing repeats of at least four units of any one of the 20 amino acids. This AAR length threshold allows one to identify proteins that contain not only long, pure homopolymeric AARs but also more fragmented repeats at a more advanced stage of their “life cycle” (Buschiazzo and Gemmell, 2006; Pelassa et al., 2014). To perform this analysis, we preliminarily scanned the Uniprot complete human proteome in search of proteins containing repeats of the different amino acids. We thus defined 20 protein groups that were identified as “polyX” groups, were X stands for the standard single letter code for one amino acid (i.e., from A to Y). These groups contain variable numbers of proteins ranging from just one, as for the polyW group, to more than 1000 proteins, as for the polyL, polyA, and polyG groups (Supplementary Table 1). We then extracted the known interactions of the proteins of each polyX group (represented schematically by *red nodes* in Figure 1A) from the whole human protein interactome reported in the BioGrid database (Stark et al., 2006), using the g:Profiler (Reimand et al., 2011) interface (Figure 1A), with the exception of the polyW group which contained only one protein. As statistical controls, for each polyX group, we also extracted in the same way the interactions of five groups of proteins selected randomly in the human proteome, each one containing the same number of proteins as the polyX group (*green nodes* in Figure 1A indicate schematically one of these control groups). We were thus able to define protein networks formed by either polyX proteins and their interactors, or by the equinumerous, randomly selected proteins and their interactors (schematized in Figure 1B as *AAR protein network* and *random protein network*, respectively). These interactomes thus contain two types of interactions, which are schematized in the lower part of Figure 1B. One type, that we called “type *a*” interactions, are between two proteins that both contain the AAR or that are both part of the list of randomly selected proteins. The other type of interactions, i.e., “type *b*,” is formed by an AAR protein with a protein that does not contain the AAR, or by a protein of the random group with another protein that is not part of the group.

**Figure 2:**
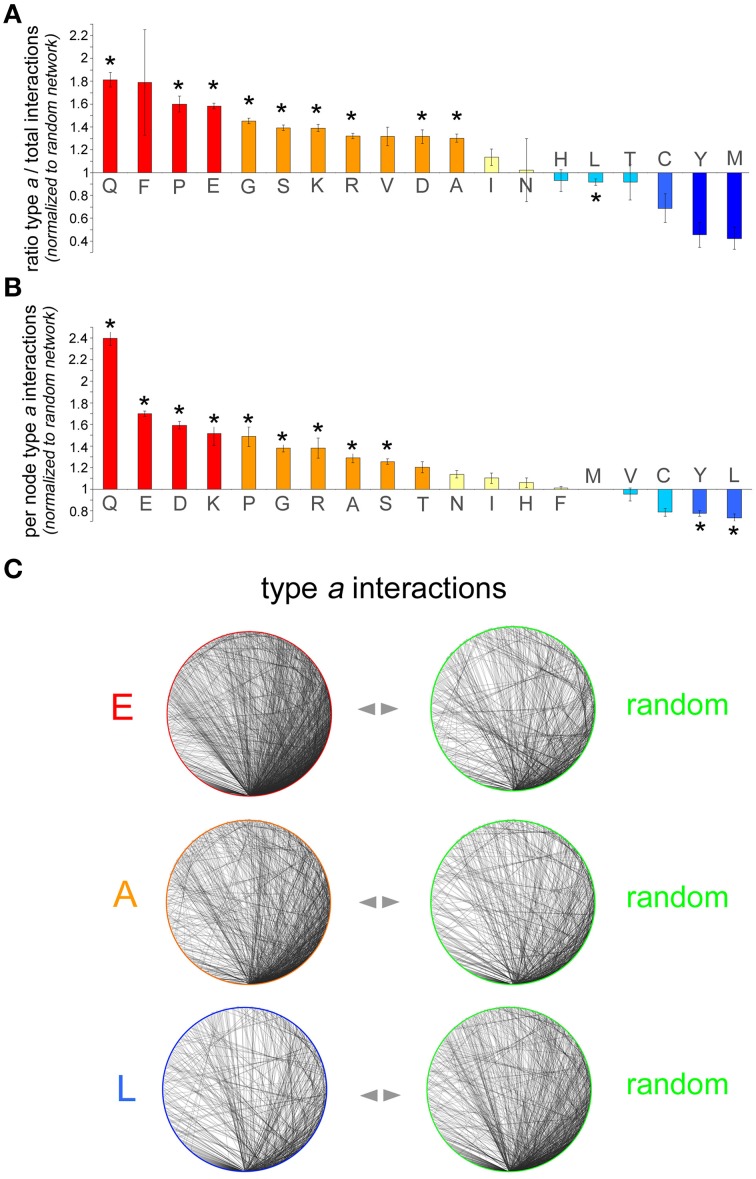
**Analysis of interaction networks formed by AAR proteins**. **(A)** Number of type *a* interactions as a proportion of the total number of interactions in the polyX protein network [i.e., type *a*/(type *a* + type *b*)] normalized to the same proportion calculated for each of the five control networks. The bars represent the average of these five normalized ratios ± SEM. Asterisks indicate polyX networks for which the proportion of type *a* interactions differs significantly from that expected by chance (*p* < 0.05, χ^2^ test with Yates' correction). **(B)** Sub-networks comprising only type *a* interactions were further analyzed and the number of per node interactions in the AAR protein sub-network was counted and normalized to the number of per node interactions in control sub-networks of random proteins. For each polyX network, this normalized value was calculated for each one of the five control networks. The graph shows, for each polyX protein group, the average normalized number of type *a* interactions per node ± SEM. **(C)** Sample sub-networks (polyE, polyA, and polyL) containing only type *a* interactions such as those that were analyzed in **(B)** (*left graphs*), with one of their respective random networks (*right graphs*). Note how the density of the polyE and polyA networks is higher than that of the corresponding control networks, whereas the density of the polyL network is lower that of the control network.

To determine whether proteins containing a certain AAR have an increased, decreased, or similar propensity to establish interactions among themselves in comparison with randomly selected proteins, we calculated two quantitative indexes by analyzing the AAR and the random protein networks (Figures 2A,B). The first index shows to what extent proteins containing a given AAR tend to interact with proteins containing the same AAR (type *a* interactions) rather than with proteins devoid of it. The second index shows the density of type *a* interactions in each AAR network.

Thus, we first calculated for each AAR protein network the number of type *a* interactions as a proportion of the total number of interactions, i.e., type *a*/(type *a* + type *b*). The same proportion was also calculated for each of the five random control networks. The value of the proportion in the AAR protein network was then normalized to the value of the proportion calculated for each of the five corresponding random protein networks. The resulting five normalized values were then averaged and are plotted in Figure 2A (mean ± SEM). This analysis revealed that, for instance, the proportion of type *a* interactions in the interactome of polyQ proteins is on average 1.81 ± 0.06 (*n* = 5) times greater than in the corresponding control networks. This difference in the distribution of type *a* and type *b* interactions between the polyQ network and the average of the control random networks was statistically significant (*p* < 0.001, χ^2^ test with Yates' correction), indicating that polyQ proteins tend to establish significantly more interactions with other polyQ proteins than expected by chance. This observation was not unique to polyQ proteins, and in fact similar results were found also for other networks of proteins containing repeats formed by other polar (polyS), charged (poly-D, -E, -K, -R) or small/cyclic (poly-A, -G, -P) amino acids. Conversely, proteins containing polyL or other hydrophobic repeats (poly-C, -M, -Y) tend to have fewer interactions with each other than expected, although this trend is statistically significant only for the polyL group. Other networks of proteins with hydrophobic AARs (poly-I and -V) display non-significant trends toward a slight increase in the proportion of type *a* interactions. The case of polyF networks is difficult to interpret due to the small number of proteins (*n* = 61) containing this repeat and to the consequently higher statistical variability that was observed in the five corresponding control networks. Finally, poly-N, -H, and -T networks did not deviate from what expected by chance in terms of type *a* connectivity.

Second, we calculated for each polyX network the average number of type *a* interactions per AAR-containing protein and we normalized this value to the corresponding value calculated for each of the five control random networks. The average of the resulting five normalized values for each AAR group is shown in Figure 2B. A One-way ANOVA analysis revealed overall significant differences among the AAR groups [*F*_(18, 76)_ = 53.69, *p* < 0.001]. Furthermore, the Dunnett *post-hoc* test, using as a control group the polyM group which has the mean value closest to 1, revealed significant differences (*p* < 0.05 in all instances) from the polyM group for all the AAR protein groups that were also significant in Figure 2A, with the addition of the polyY group.

This analysis showed, for example, that polyQ proteins establish 2.39 ± 0.06 (*n* = 5) times more interactions with other polyQ proteins than expected by chance. Also other networks of proteins containing repeats of polar (polyS), charged (poly-D, -E, -K, -R), or small/cyclic (poly-A, -G, -P) amino acids display 1.25–1.7 times more per-node interactions than expected by chance, as also illustrated in the sample networks shown in Figure 2C. This figure illustrates how, for instance, networks of polyE or polyA proteins display a higher density of type *a* connections than the corresponding control networks. Again, networks of proteins containing certain hydrophobic AARs (poly-L, -Y) displayed a significantly lesser number of per node connections than expected by chance, as one can also appreciate visually in Figure 2C which shows how the density of the polyL network is lower than that of a random network. Finally, the density of type *a* connections in poly-T, -N, -I, -H, -F, -M, and -V networks did not significantly differ from that of the corresponding control networks. Taken together, these findings show a substantial concordance of the two indexes that we used to characterize the type *a* connectivity of the AAR protein networks. In fact, we observed a significant correlation between the two indexes of the 19 AAR groups (*r* = 0.69, *n* = 19, *p* < 0.01). Thus, AAR groups in which the first index is high and statistically significant tend to have also higher values for the second index (e.g., polyQ, polyP, polyE), and this general concordance of the two indexes strengthens the conclusion that these AARs are associated with a higher degree of interactivity among proteins that contain them. Conversely, in some particular cases like that of polyF, even though the first index is high, but not significantly, the second index is close to the value expected by chance, thus indicating overall that the presence of this repeat is not associated with a greater connectivity between the proteins that contain it.

Taken together, these findings indicate that the presence of certain AARs in protein networks associates with a higher degree of connectivity. These AARs are those formed by certain polar (poly-Q, -S), charged (poly-D, -E, -K, -R), or small/cyclic (poly-A, -G, -P) amino acids, suggesting that these repeats themselves, or protein domains they co-occur with, or they are found within, may promote protein-protein interactions and the formation of interaction networks. Conversely, the presence of certain hydrophobic repeats like polyL and polyY in proteins seems to disfavor the formation of interaction networks, possibly owing to the fact that these repeats are often found in transmembrane domains that sequester proteins in membranes (see Section Discussion).

### Possible roles of AARs in protein-protein, -nucleic acid, and -lipid interactions

**Figure 3:**
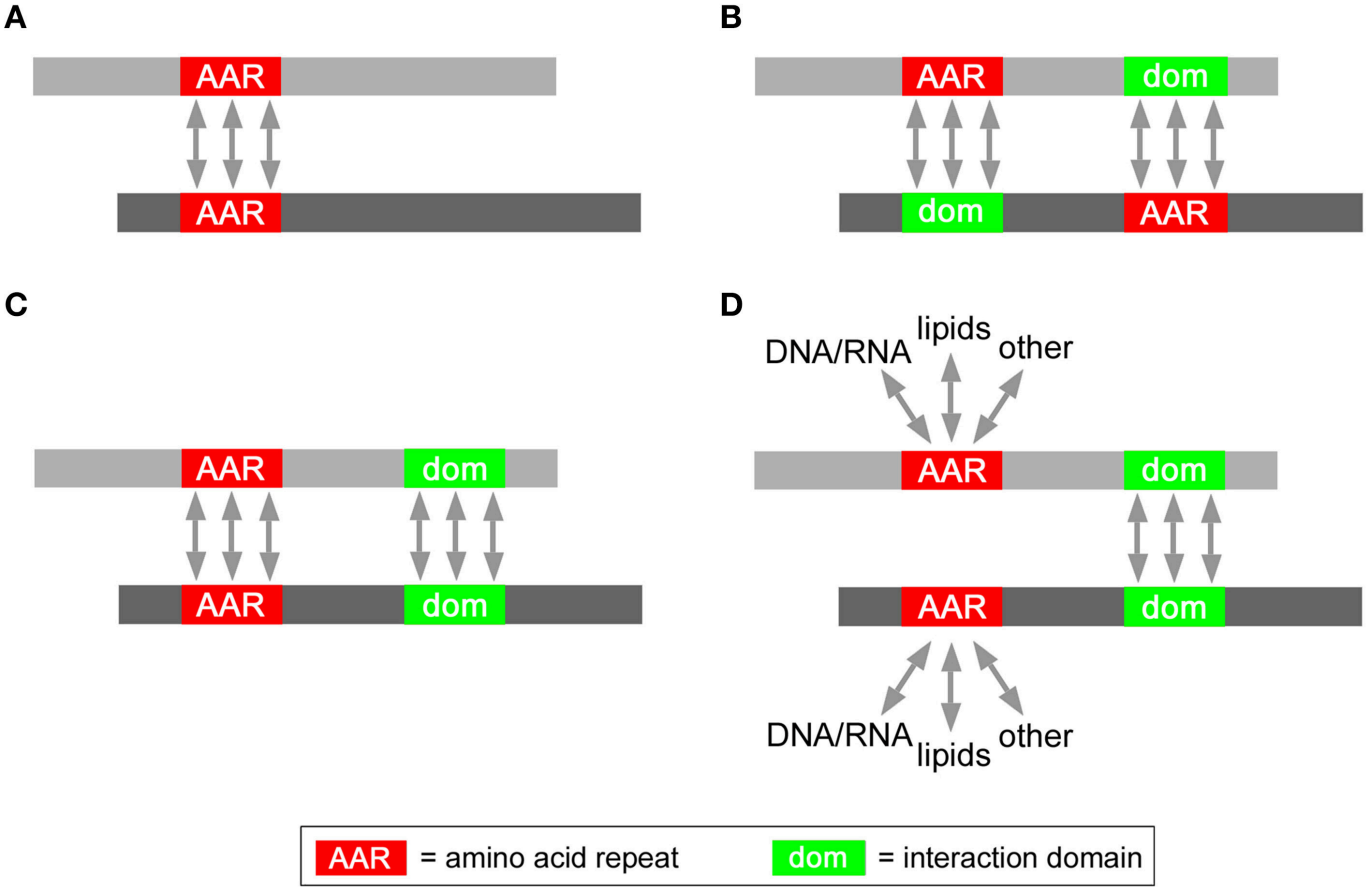
**Possible roles of AARs in protein interaction networks**. Schematic representation of possible modes of interaction between two AAR-containing proteins (*gray bars*) mediated either by **(A)** homotypic AAR-AAR contacts, or **(B)** by heterotypic AAR-interaction domain contacts, or **(C)** homotypic AAR-AAR and domain-domain contacts, or by **(D)** homotypic domain-domain interactions.

In principle, several non-exclusive structural mechanisms (Figure 3) may underlie the enhanced mutual interaction propensity of proteins containing certain AARs. Interestingly, some of these possibilities have already been demonstrated experimentally, while others will need to be further investigated in biological experiments. In the simplest case (Figure 3A), AARs themselves may be the structural mediators of protein-protein interactions. For instance, polyQ and polyA repeats can mediate protein interactions and oligomerization by forming coiled-coil structures (Fiumara et al., 2010; Pelassa et al., 2014). Another possibility (Figure 3B) is that AARs in one protein interact with another structural domain of another protein, as known for the case of proline-rich stretches forming polyproline-II (PP-II) structures which can be bound by SH3 domains (Yu et al., 1994). The enrichment of such AAR-targeting interaction domains in the same proteins containing the AAR may explain the increased tendency of such proteins to interact with each other. In some polyX networks (Figure 3C) interactions may also be promoted synergistically by AARs and other conventional protein-protein interaction domains. For instance, polyQ and/or polyA repeats and flanking sequences with coiled coil propensity may co-operate in protein interactions (Fiumara et al., 2010; Pelassa et al., 2014). In principle (Figure 3D), certain AARs may even not have a direct role in promoting the interactions between proteins in which they are present (Figure 3D). In this case, the interaction would be mediated by conventional protein-protein interaction domains that are overrepresented in the AAR protein group. AARs in this scenario may be involved in interactions with other cellular components like nucleic acids and lipids, or may have other roles unrelated to protein interaction. A possible example of this scenario may be that of proteins containing both charged repeats like polyK and conventional CC domains. In this case, while coiled coils could mediate the protein-protein interactions, the charged repeats may mediate instead interactions with negatively charged surfaces such as the phospolipid bilayer.

Biological experiments will be ultimately necessary for discriminating between these possibilities for the different polyX protein groups. As a first step in this direction, however, it may be important to determine initially, through a systematic proteome-wide analysis, which protein domains are significantly overrepresented in each polyX protein group. These domains may in fact be responsible, together with AARs or by themselves, for the increased mutual interaction propensity of AAR-containing proteins. Thus, this analysis may ultimately guide the biological exploration of the role of AARs in protein interaction networks by indicating which AAR/domain associations are most likely to determine an increase in protein interactivity such as we observe in certain AAR-containing protein groups.

### Co-occurrence in proteins of AARs and protein-protein interaction domains

**Figure 4:**
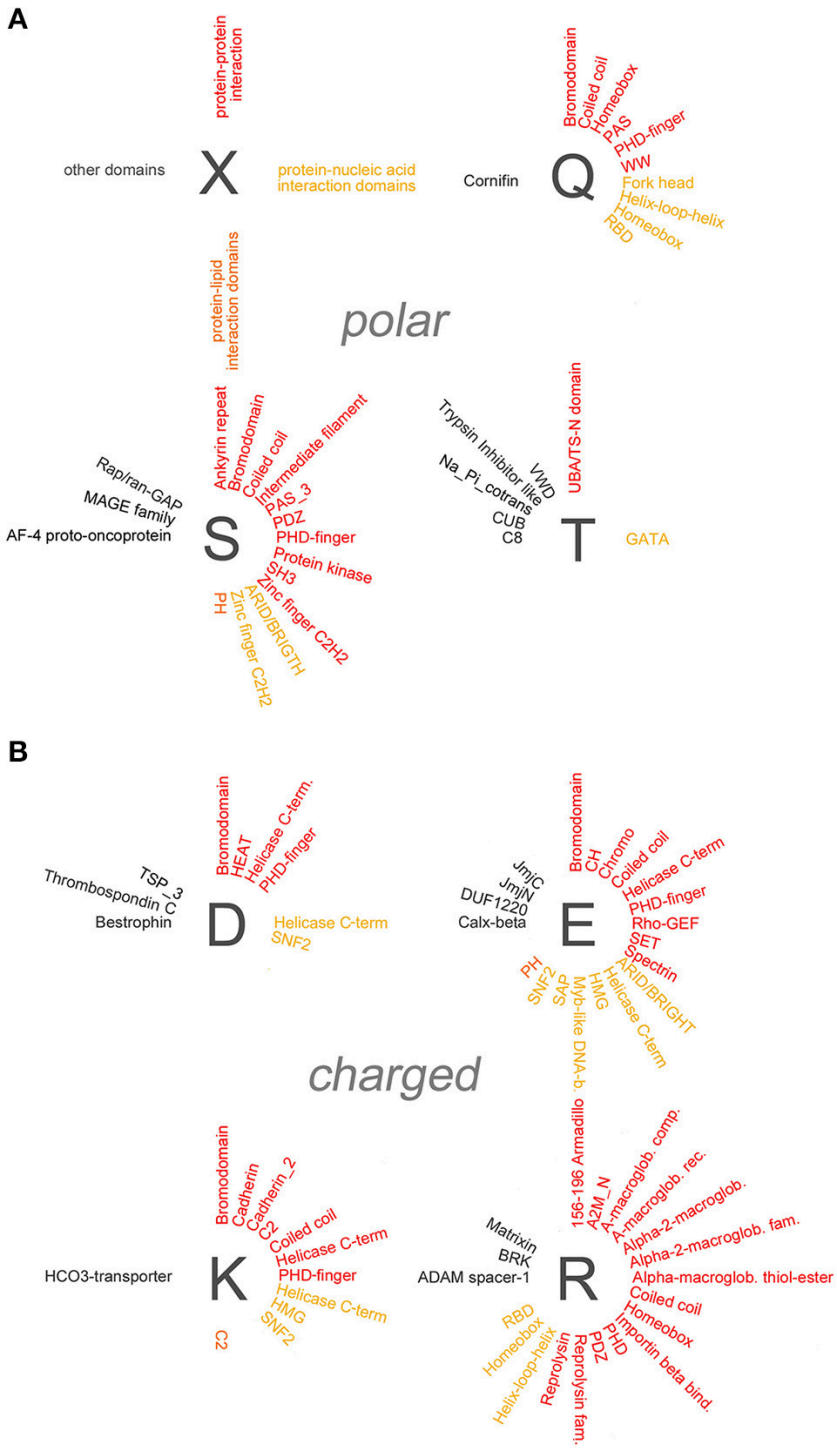
**Overrepresentation of protein-protein, -nucleic acid, and -lipid interaction domains in proteins containing polar and charged AARs**. **(A)** Schematic radial representation of the protein domains that are significantly enriched in protein groups containing AARs formed by the polar amino acids indicated by the *dark gray letters*. The legend in the *upper left quadrant* (X) shows how protein-protein interaction domains are indicated in *red*, protein-nucleic acid interaction domains in *dark yellow*, protein-lipid interaction domains in *orange*, and domains with other or unknown functions in *black.* **(B)** As in **(A)** for groups of proteins containing charged AARs.

**Figure 5:**
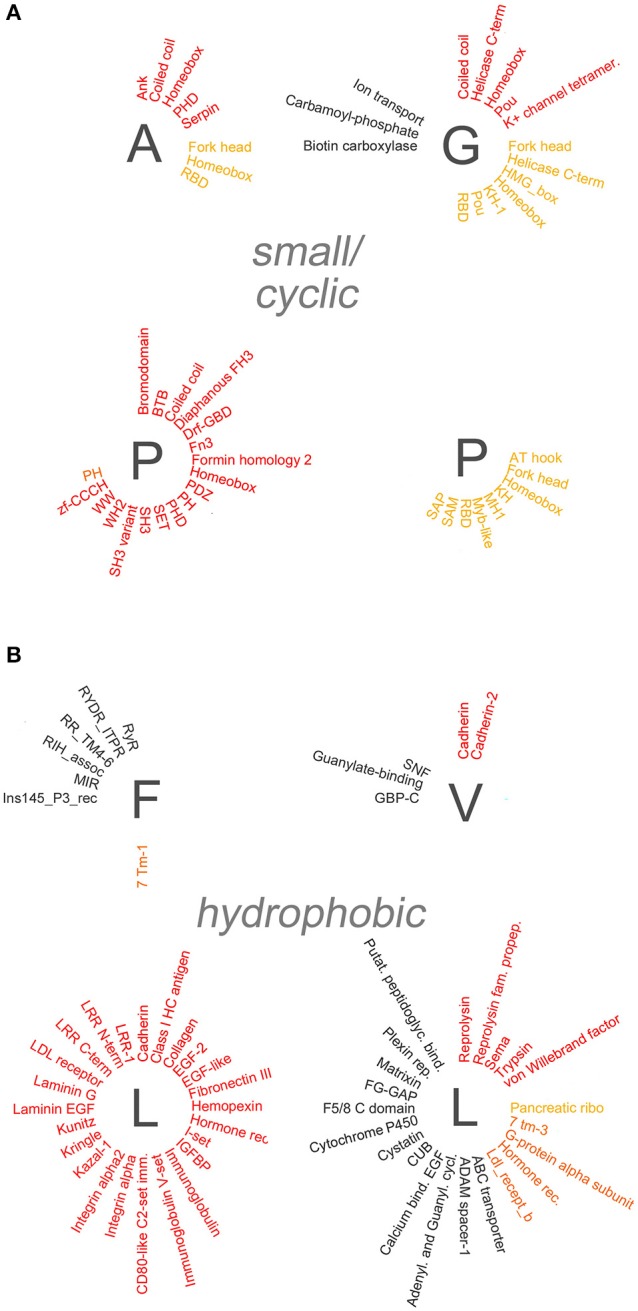
**Overrepresentation of protein-protein, -nucleic acid, and -lipid interaction domains in proteins containing small/cyclic and hydrophobic AARs**. Schematic radial representation, as in Figure 4A, of the protein domains that are significantly enriched in protein groups containing AARs formed by small/cyclic **(A)** or hydrophobic **(B)** amino acids.

To determine whether specific protein domains are overrepresented in the different groups of polyX proteins, we analyzed statistically their domain composition using the DAVID database (Dennis et al., 2003). Specifically, we searched for protein domains which are enriched in each of the polyX protein lists (except for polyW) using a stringent statistical criterion (*p* < 0.05 after applying the Benjamini-Hochberg adjustment). Overall, this analysis revealed the overrepresentation of multiple types of protein domains in most polyX protein groups, with the exception of the poly-C, -M, -N, and -Y groups. An exhaustive list of these domains is reported in the Supplementary Table 2 and is represented graphically in Figures 4, 5 and in Supplementary Figure 1. We categorized these domains in four groups, i.e., (i) protein-protein, (ii) protein-nucleic acid, (iii) protein-lipid interaction domains, and (iv) domains involved in other functions or with unclear function. Some domains belong to more than one category as they have been shown to mediate multiple functions (e.g., DNA binding and protein-protein interactions).

Several polyX groups of proteins displayed selective enrichments of domains belonging to these four categories, and individual domains can co-occur with multiple types of AARs. The highest number of significant enrichments of protein-protein interaction domains was observed in the poly-S, -E, -K, -R-, -P, and -L groups. Conversely, using the stringent criteria that were adopted, no significant overrepresentation of domains was observed in the poly-C, -M, -N, and -Y groups.

A paradigmatic case of co-occurrence of AARs and protein-protein interaction domains is that of coiled coil domains. These structural domains are indeed significantly overrepresented in proteins containing polar (poly-Q, -S), charged (poly-E, -K, -R), and small/cyclic (poly-A, -G, -P) AARs. Interestingly, in some of these cases (polyQ, polyA) the AARs themselves are known to form coiled coil structures often as part of conventional coiled coil domains (Fiumara et al., 2010; Pelassa et al., 2014), and the same may be in principle possible for short poly-S, -E, -K, and -R stretches when embedded in conventional coiled coil sequences. On the other hand, polyP and polyG stretches form other types of structures (Adzhubei et al., 2013) and their observed co-occurrence with coiled coil domains in the same proteins may not be obviously due to overlap between the AAR and the domain but to other functional reasons. These observations indicate that, at least in certain cases, the observed enrichments of certain domains in some polyX protein groups may result from the at least partial overlap of the repeat and the domains (see the last section of the Results and the Supplementary Table 3). Besides coiled coils, other domains such as the bromodomain, which binds acetylated lysine residues on histone proteins, and the PDZ domain, which is commonly found in signaling proteins, are similarly overrepresented in multiple polyX protein groups (poly-D, -E, -K, -P, -Q, -S and poly-P, -R, -S, respectively). Other protein-protein interaction domains tend to co-occur more specifically only with a few polyX protein groups. For example, SH3 domains are enriched only in the poly-P and -S groups, whereas HEAT domains, helical structures involved in intracellular transport, are overrepresented only in the polyD protein group. Interestingly, some nucleic acid-binding domains which also function as protein-protein interaction domains, like the Homeobox domain, are overrepresented especially in protein containing certain polar (polyQ) charged (polyH), or small/cyclic (poly-A, -G, -P) repeats. PolyL proteins represent a quite unique case as they contain a high number of protein-protein interaction domains, mostly associated with trans-membrane or secreted proteins. Such abundance of overrepresented domains in the polyL group may be likely explained by the fact that polyL repeats are often found in signal peptide and transmembrane regions which are characteristic of proteins targeted to the secretory pathway or to cellular membranes with ligand/receptor functions (see Section Discussion). Notably, protein-protein interaction domains are conversely rarely co-occurring with other hydrophobic AARs.

### Co-occurrence in proteins of AARs and protein-nucleic acid interaction domains

Domains known to mediate protein-nucleic acid interactions are frequently overrepresented in different polyX protein groups. In particular, multiple DNA-binding domains (e.g., Homeobox, Fork head, helix-loop-helix (HLH), helicase domains) co-occur in proteins with charged, polar, and small/cyclic AARs. The particular enrichment of DNA binding domains in groups of proteins containing charged repeats may reside in the capacity of charged AARs to bind DNA and chromatin components such as the histones (e.g., Dean, 1983; DeRouchey et al., 2013). Thus, charged AARs may have synergistic roles with DNA binding domains in driving the interaction of proteins with the nuclear genetic material. Instead the co-occurrence of this type of domain with hydrophobic AARs is quite exceptional. Interestingly, RNA-binding domains (RBD) are particularly enriched in protein groups containing polar (polyQ) and small/cyclic (poly-A, -G, -P) AARs, but not, at variance with DNA-binding domains, in protein groups with charged AARs, except for the group containing polyR repeats which may favor RNA binding (e.g., Nam et al., 2001).

### Co-occurrence in proteins of AARs, protein-lipid interaction domains, and other domains

We also found evidence for the overrepresentation of some lipid-binding domains in some polyX proteins groups. Rodopsin-like and class C G-protein-coupled receptors are overrepresented in polyI, polyF, and polyL proteins. PolyI repeats also co-occur with synaptobrevin domains. In most cases the hydrophobic repeats lie within the domains themselves as part of transmembrane regions (Supplementary Table 3). Two other lipid-binding domains are enriched in non-hydrophobic polyX protein groups. The CH2 domain targets proteins to membranes and is overrepresented in proteins containing polyK repeats, which may indeed also contribute to phospholipid binding (e.g., Reuter et al., 2009), whereas the pleckstrin homology (PH) domains, which bind phosphoinositides, are overrepresented in the poly-E, -S, and -P protein groups.

Taken together, these findings indicate that specific patterns of co-occurrence exist in proteins between AARs and protein domains that mediate interactions with other proteins, nucleic acids, and lipids. These domains, together with the AARs themselves, may contribute to shaping interactomes as illustrated in Figure 3.

### Overlap of AARs and protein domains

As observed for polyQ and polyA repeats in coiled coil domains (Fiumara et al., 2010; Pelassa et al., 2014), the possibility exists that certain repeats may not only co-occur with interaction domains in the same proteins but may also be embedded within these domains. To determine whether this is the case, we verified in the NCBI Conserved Domains Database (CDD) (available at http://www.ncbi.nlm.nih.gov/Structure/cdd/cdd.shtml), for each significant AAR-domain co-occurrence, in which proportion of proteins the AAR and the domain overlap for at least four residues. We found that in 148 out of 189 significant cases of AAR-domain co-occurrence no overlap exists between AARs and the domains that co-occur with them in the same proteins. However, for 41 cases of AAR-domain co-occurrence there is some sign of overlap between repeats and domains. In 21 cases, the overlap is observed in between 25 and 100% of the proteins containing the AAR-domain combination in the CDD database (Supplementary Table 3). For example, short polyR repeats were observed within the homeobox domains in 15 out of 22 proteins (i.e., 68%) that contain the polyR-homeobox association, and within the HLH domain in 8 out of 16 proteins (i.e., 50%) containing the polyR-HLH association. These observations indicate that AARs can be part of protein interaction domains and possibly play a functional role in them.

## Discussion

The results of our analyses indicate overall that the presence of certain types of AARs in protein networks is associated with a significantly increased protein-protein connectivity, and that significant patterns of co-occurrence, and in some cases overlap, exist between AARs and conventional protein interaction domains. These findings suggest that different types of AARs may play a generalized, combinatorial role in shaping protein interaction networks together with conventional protein-protein interaction domains they co-occur with.

### Structural and functional roles of AARs in protein interactions

We found that proteins that contain a variety of polar (polyQ and polyS), charged (poly-D, -E, -K, -R), and small/cyclic (poly-A, -G, -P) repeats show a greater tendency to interact among themselves, in comparison with proteins devoid of these AARs. This is also paralleled, in the networks formed by proteins bearing these “interaction-enhancing” AARs, by a higher number of AAR-containing interaction partners per AAR protein. The opposite phenomena were observed for the protein groups containing certain hydrophobic AARs, like polyL and polyY, whereas for other AAR protein groups there was no evidence for statistically significant changes in type *a* connectivity. The observed reduction in connectivity among proteins containing polyL and polyY may likely result from the fact that these hydrophobic repeats are mostly part of transmembrane domains and signal peptides (Hikita and Mizushima, 1992; Zhou et al., 2001). In fact, the localization in membranes may limit relatively the possibility of proteins to interact with other proteins in the same membrane compartment, while preserving the possibility of interaction with intra- and extra-cellular (or intra- and extra-luminal, in the case of organelles) proteins. On the other hand, the increased propensity for mutual interactions observed among proteins containing poly-Q, -S, -D, -E, -K, -R, -A, -G, and -P repeats may have several possible, and not mutually exclusive, explanations, as schematized in Figure 3. Experimental evidence already indicates that at least polyQ and polyA repeats may directly mediate protein-protein interactions by forming coiled coil structures that can also interact with conventional, non-repetitive coiled coils (Fiumara et al., 2010; Schaefer et al., 2012; Pelassa et al., 2014). This type of interaction between proteins mediated directly by homotypic AAR-AAR contacts may not be a universal phenomenon in the polyX interactomes with enhanced connectivity that we have analyzed. In fact, polyproline-II structures in proline-rich and polyP-containing proteins are known to establish heterotypic interactions with SH3 domains (Yu et al., 1994) which we find being enriched precisely in the same polyP protein group. Thus, the enhanced connectivity observed in some AAR protein networks may result from the enrichment in them of interaction domains capable of AAR binding. While this possibility needs to be tested experimentally for the different AARs, our analyses identified a relatively restricted subset of significantly enriched domains in each polyX protein group that may play a role similar to that of SH3 domains in the interactome of polyP proteins. AARs and conventional protein interaction domains may also cooperate in mediating the binding of proteins to other proteins or to nucleic acids and cellular membranes. This seems particularly plausible for charged AARs. Charged AARs can indeed bind DNA and RNA (e.g., Dean, 1983; Nam et al., 2001; DeRouchey et al., 2013) and may therefore cooperate with sequence-specific DNA- or RNA-binding domains in stabilizing protein interactions with nucleic acids. Charged AARs can also bind histones and may cooperate with DNA-binding domains within the same protein that bind histone-associated DNA. Positively charged AARs can also bind phospholipids (Schwieger and Blume, 2007; Reuter et al., 2009) and we found indeed evidence of a significant overrepresentations of CH2 lipid binding domains in polyK proteins.

### Physiological and pathological roles of AARs in shaping protein interactomes

Taken together these observations indicate that, given their widespread presence in proteomes and their frequent co-occurrence with protein interaction domains, polyQ -S, -D, -E, -K, -R, -A, -G, and -P AARs may play a significant, generalized role in shaping protein interaction networks. Interestingly, most of these interaction-enhancing AARs can form defined secondary and supersecondary structures like α-helical coiled coil structures in the case of polyQ and polyA repeats (Fiumara et al., 2010; Pelassa et al., 2014), and polyproline II (PP-II) and polyglycine II (PG-II) structures (e.g., Adzhubei et al., 2013) in the case of polyP and polyG repeats, respectively. Also PolyE and polyK repeats can form helical structures in a pH-dependent manner (Inoue et al., 2005; Mirtič and Grdadolnik, 2013), and it is thus conceivable that short repeats of glutamate or other charged amino acids may well be incorporated into defined protein structures. Thus, AARs may favor the formation of protein interactions not only as intrinsically disordered domains through the formation of “fuzzy” complexes (van der Lee et al., 2014) but also through the formation of defined secondary structures, similar to conventional, non-repetitive interaction domains. This conclusion is also supported by our observation that conventional protein interaction domains can contain short AARs within them, which are likely to take part in some aspect of their structure/function.

Different AARs can co-occur in the same protein groups, as observed for polyQ and polyA repeats (Pelassa et al., 2014), and for other AARs (Pelassa and Fiumara, unpublished observations). These observations, together with the existence of specific patterns of co-occurrence of AARs and protein interaction domains, strongly suggest the existence of a combinatorial protein interaction code defined by the variable co-occurrence in different protein groups of multiple types of AARs and of conventional interaction domains. These domains can indeed establish homotypic AAR-AAR and domain-domain interactions, as well as heterotypic AAR-domain and domain-domain interactions. Thus, the combination in one protein of AARs and of various types of interaction domains can finely tune the specificity and stability of the binding of the protein to other proteins, but also to nucleic acids and to phospholipids in membranes. Our observations identify overall a number of potentially relevant AAR-domain co-occurrences whose functional relevance ought to be experimentally tested in different biological contexts, such as transcriptional and translational regulation, protein trafficking, *et cetera*. Biological experiments guided by our findings may ultimately help to define the exact role and the relative contribution of AARs and of co-occurring interaction domains in shaping both the physiological interactomes in the human proteome and the aberrant, pathological protein interaction networks that are established in polyQ or polyA expansion diseases.

Thus, in conclusion, the results of our analyses contribute proteome-wide quantitative evidence supporting the existence of physiological, structural and functional roles of AARs, and pave the way to the informed biological dissection of AAR-mediated protein interaction networks in health and disease.

## Materials and methods

### Datasets

The complete reference proteome of *Homo sapiens* was retrieved from the Uniprot database (www.uniprot.org) in FASTA format without isoforms. The proteins containing AARs of at least four units were identified using *ad hoc* Perl scripts as in Pelassa et al. (2014). The domain composition of the proteins of interest was derived performing batch searches on the NCBI CCD website (available at http://www.ncbi.nlm.nih.gov/Structure/cdd/wrpsb.cgi). Necessary conversions of the different protein identifiers found in the different databases were performed using the DAVID (https://david.ncifcrf.gov/) or Biomart (www.biomart.org) databases.

### Definition and analysis of PolyX protein interactomes and control random interactomes

Protein interaction networks formed by proteins containing a given AAR and their interactors were extracted form the BioGrid database of protein-protein interactions using the g:Profiler web interface (available at http://biit.cs.ut.ee/gprofiler/), deselecting the “significant only” option so that all interactions could be downloaded in a tab-delimited text files. Control networks formed by proteins selected at random in the human proteome and their interactors were obtained in the same way. Randomness in the selection of the proteins was achieved by using a random number generator to select protein IDs from a complete list of all the human protein IDs ordered as elements of an array. In particular, we reiteratively used the Perl “rand” function to select sets of random elements of the desired numerosity from the elements of this array. The files derived from g:Profiler for both AAR and random networks were then analyzed with *ad hoc* Perl scripts in order to identify and quantify “type *a*” and “type *b*” interactions (see Section Results).

### Analysis of the overrepresentation of protein domains in PolyX protein groups

The protein domains that are overrepresented in the polyX protein groups were identified using the DAVID database. We searched, using the “Protein domains” selection menu, for “Pfam” domains enriched with a Benjamini score < 0.05. Coiled coil domains were identified, using the “Functional categories” selection menu, searching for “SP_PIR_KEYWORDS.”

### Analysis of the overlap between AARs and protein domains

The overlap between AARs and conventional domains in proteins was determined using *ad hoc* Perl scripts. These scripts compared for each protein the relative positions of the AARs and of protein domains whose positions were derived from the NCBI CDD database (see the Section Datasets above). The overlap of coiled coil domains with polyQ and polyA repeats was shown previously (Fiumara et al., 2010; Schaefer et al., 2012; Pelassa et al., 2014), and here we did not analyze further the coiled coil/AAR overlap.

### Graphs

Bar graphs were generated using Excel software (Microsoft). Network graphs were generated using CytoScape software (available at www.cytoscape.org) selecting the “degree sorted circle layout.” Other schematic representations and figures were generated using Photoshop Elements 11 software (Adobe).

### Data analysis and statistics

Data were processed and analyzed statistically using Excel (Microsoft) and SPSS 21 (IBM) software. Appropriate statistical tests were performed as indicated in the text and *p* < 0.05 was considered as statistically significant in all instances.

### Conflict of interest statement

The authors declare that the research was conducted in the absence of any commercial or financial relationships that could be construed as a potential conflict of interest.

## Supplementary material

Supplementary Table 1 **Number of proteins in the 20 polyX groups**.

Supplementary Table 2 **Protein domains overrepresented in polyX protein groups**.

Supplementary Table 3 **Overlap between AARs and protein domains**.

Supplementary Figure 1 **Overrepresentation of protein-protein, -nucleic acid, and -lipid interaction domains in proteins containing polyH and polyI AARs**.
