# Supplementary material for: Differential Occurrence of Interactions and Interaction Domains in Proteins Containing Homopolymeric Amino Acid Repeats
Source: Front Genet. 2015 Dec 18;6:345. doi: 10.3389/fgene.2015.00345 (PMC4683181; doi:10.3389/fgene.2015.00345)
Supplement: Supplementary Table 1 — Number of proteins in the 20 polyX groups. [file Table1.pdf]

Supplementary table 1 – *Number of proteins in the 20 polyX groups*

AAR	Number of proteins containing the AAR in the complete human proteome (proteome size = 20266 proteins)
polyA	1576
polyC	61
polyD	307
polyE	1769
polyF	61
polyG	1071
polyH	130
polyI	72
polyK	697
polyL	2001
polyM	9
polyN	58
polyP	1562
polyQ	466
polyR	666
polyS	1799
polyT	238
polyV	246
polyW	1
polyY	30
