# Supplementary material for: Differential Occurrence of Interactions and Interaction Domains in Proteins Containing Homopolymeric Amino Acid Repeats
Source: Front Genet. 2015 Dec 18;6:345. doi: 10.3389/fgene.2015.00345 (PMC4683181; doi:10.3389/fgene.2015.00345)
Supplement: Supplementary Table 2 — Protein domains overrepresented in polyX protein groups. [file Table2.pdf]

Supplementary table 2 – *Protein domains overrepresented in polyX protein groups*

The table indicates the protein domains, identified by their Pfam ID (*second column from the left*), that are significantly overrepresented in the AAR-containing protein groups (*first column*), as determined using the DAVID database (https://david.ncifcrf.gov/) searching for “Pfam” domains, with a p<0.05 after the Benjamini-Hochberg adjustment. The functions of each domain, as derived from the Pfam database (http://pfam.xfam.org/) or from the literature (e.g. Xia et al., 2008, Genome Res., 18:1500-8), are indicated by *cross marks* in the four columns on the right, where *Protein* indicates a role of the domain in protein-protein interactions, *Nucleic acid* indicates a role of the domain in protein-nucleic acid interactions, *Lipid* indicates a role of the domain in protein-lipid interactions, also through extensive transmembrane regions, and *Other* indicates other type of functions or unknown functions. These attributions of the domains to the different categories are purely indicative and do not exclude other roles for each domain. The overrepresentation of coiled coil domains, which can be protein-protein interaction domains, was determined by using the DAVID database selecting the “Functional categories” menu, searching for “SP_PIR_KEYWORDS.”

AAR	Pfam ID	Protein	Nucleic acid	Lipid	Other
A	PF00023:Ank	X			
A	PF00046:Homeobox domain	X	X		
A	PF00076:RBD		X		
A	PF00079:Serpin	X			
A	PF00250:Fork head domain		X		
A	PF00628:PHD	X			
A	SP_PIR_KEYWORDS:coiled coil	x			
D	PF00176:SNF2 family N-terminal domain		X		
D	PF00271:Helicase conserved C-terminal domain	X	X		
D	PF00439:Bromodomain	X			
D	PF00628:PHD-finger	X			
D	PF01062:Bestrophin				X
D	PF02412:TSP_3				X
D	PF02985:HEAT	X			
D	PF05735:Thrombospondin C-terminal region				X
E	PF00169:PH domain			X	
E	PF00176:SNF2 family N-terminal domain		X		
E	PF00249:Myb-like DNA-binding domain		X		
E	PF00271:Helicase conserved C-terminal domain	X	X		
E	PF00307:CH	X			
E	PF00385:Chromo	X			
E	PF00435:Spectrin	X			
E	PF00439:Bromodomain	X			
E	PF00505:HMG		X		
E	PF00621:RhoGEF domain	X			
E	PF00628:PHD-finger	X			
E	PF00856:SET domain	X			
E	PF01388:ARID/BRIGHT DNA binding domain		X		
E	PF02037:SAP domain		X		

E	PF02373:JmjC domain				X
E	PF02375:JmjN				X
E	PF03160:Calx-beta				X
E	PF06758:DUF1220				X
E	SP_PIR_KEYWORDS:coiled coil	x			
F	PF00001:7tm_1			X	
F	PF01365:RYDR_ITPR				X
F	PF02026:RyR				X
F	PF02815:MIR				X
F	PF06459:RR_TM4-6				X
F	PF08454:RIH_assoc				X
F	PF08709:Ins145_P3_rec				X
G	PF00013:KH_1		X		
G	PF00046:Homeobox	X	X		
G	PF00076:RBD		X		
G	PF00157:Pou	X	X		
G	PF00250:Fork head domain		X		
G	PF00271:Helicase conserved C-terminal domain	X	X		
G	PF00289:Carbamoyl-phosphate synthase L chain, N-terminal domain				X
G	PF00505:HMG_box		X		
G	PF00520:Ion transport protein				X
G	PF02214:K+ channel tetramerization domain	X			
G	PF02785:Biotin carboxylase C-terminal domain				X
G	SP_PIR_KEYWORDS:coiled coil	x			
H	PF00046:Homeobox	X	X		
H	PF00157:Pou	X	X		
H	PF03131:bZIP_Maf	X	X		
H	PF08383:Maf_N	X	X		
I	PF00001:7 transmembrane receptor			X	
I	PF00957:Synaptobrevin	X			
I	PF02760:HIN-200/IF120x domain				X
K	PF00028:Cadherin	X			
K	PF00168:C2 domain	X		X	
K	PF00176:SNF2 family N-terminal domain		X		
K	PF00271:Helicase conserved C-terminal domain	X	X		
K	PF00439:Bromodomain	X			
K	PF00505:HMG		X		
K	PF00628:PHD-finger	X			
K	PF00955:HCO3- transporter family				X
K	PF07565:Band_3_cyto			X	
K	PF08266:Cadherin_2	X			
K	SP_PIR_KEYWORDS:coiled coil	x			
L	PF00003:7tm_3			X	
L	PF00005:ABC transporter				X
L	PF00008:EGF-like domain	X			

L	PF00014:Kunitz/Bovine pancreatic trypsin inhibitor domain	X			
L	PF00028:Cadherin	X			
L	PF00031:Cystatin				X
L	PF00041:Fibronectin type III domain	X			
L	PF00045:Hemopexin	X			
L	PF00047:Immunoglobulin domain	X			
L	PF00050:Kazal_1	X			
L	PF00051:Kringle domain	X			
L	PF00053:Laminin_EGF	X			
L	PF00057:Low-density lipoprotein receptor domain class A	X			
L	PF00058:Ldl_recept_b			X	
L	PF00067:Cytochrome P450				X
L	PF00074:Pancreatic ribonuclease		X		
L	PF00089:Trypsin	X			
L	PF00092:von Willebrand factor type A domain	X			
L	PF00129:Class I Histocompatibility antigen, domains alpha 1 and 2	X			
L	PF00211:Adenylate and Guanylate cyclase catalytic domain				X
L	PF00219:IGFBP	X			
L	PF00357:Integrin_alpha	X			
L	PF00413:Matrixin				X
L	PF00431:CUB domain				X
L	PF00503:G-protein alpha subunit			X	
L	PF00560:LRR_1	X			
L	PF00754:F5/8 type C domain				X
L	PF01391:Collagen	X			
L	PF01403:Sema	X			
L	PF01421:Reprolysin	X			
L	PF01437:Plexin repeat				X
L	PF01462:Leucine rich repeat N-terminal domain	X			
L	PF01463:Leucine rich repeat C-terminal domain	X			
L	PF01471:Putative peptidoglycan binding domain				X
L	PF01562:Reprolysin family propeptide	X			
L	PF01839:FG-GAP				X
L	PF02210:Laminin G domain	X			
L	PF02793:Hormone receptor domain			X	
L	PF05986:ADAM_spacer1				X
L	PF07645:Calcium binding EGF domain				X
L	PF07679:I-set	X			
L	PF07686:Immunoglobulin V-set domain	X			
L	PF07974:EGF_2	X			
L	PF08205:CD80-like C2-set immunoglobulin domain	X			
L	PF08441:Integrin_alpha2	X			
P	PF00013:KH domain		X		
P	PF00018:SH3 domain	X			
P	PF00041:fn3	X			

P	PF00046:Homeobox domain	X	X		
P	PF00076:RBD		X		
P	PF00169:PH domain			X	
P	PF00249:Myb-like DNA-binding domain		X		
P	PF00250:Fork head domain		X		
P	PF00397:WW domain	X			
P	PF00439:Bromodomain	X			
P	PF00536:SAM domain		X		
P	PF00595:PDZ domain	X			
P	PF00628:PHD	X			
P	PF00642:zf-CCCH	X			
P	PF00651:BTB	X			
P	PF00856:SET domain	X			
P	PF02037:SAP domain		X		
P	PF02178:AT hook motif		X		
P	PF02181:Formin Homology 2 Domain	X			
P	PF02205:WH2 motif	X			
P	PF03165:MH1		X		
P	PF06367:Diaphanous FH3 Domain	X			
P	PF06371:Drf_GBD	X			
P	PF07653:Variant SH3 domain	X			
P	SP_PIR_KEYWORDS:coiled coil	x			
Q	PF00010:Helix-loop-helix DNA-binding domain		X		
Q	PF00046:Homeobox domain	X	X		
Q	PF00076:RBD		X		
Q	PF00250:Fork_head		X		
Q	PF00397:WW	X			
Q	PF00439:Bromodomain	X			
Q	PF00628:PHD-finger	X			
Q	PF00989:PAS	X			
Q	PF02389:Cornifin				X
Q	SP_PIR_KEYWORDS:coiled coil	x			
R	PF00010:Helix-loop-helix DNA-binding domain		X		
R	PF00046:Homeobox domain	X	X		
R	PF00076:RBD		X		
R	PF00207:Alpha-2-macroglobulin family	X			
R	PF00413:Matrixin				X
R	PF00514:156-196 Armadillo	X			
R	PF00595:PDZ domain	X			
R	PF00628:PHD	X			
R	PF01421:Reprolysin	X			
R	PF01562:Reprolysin family propeptide	X			
R	PF01749:Importin beta binding domain	X			
R	PF01835:A2M_N	X			
R	PF05986:ADAM_spacer1				X
R	PF07533:BRK domain				X

R	PF07677:A-macroglobulin receptor	X			
R	PF07678:A-macroglobulin complement component	X			
R	PF07703:Alpha-2-macroglobulin family N-terminal region	X			
R	PF10569:Alpha-macro-globulin thiol-ester bond-forming region	X			
R	SP_PIR_KEYWORDS:coiled coil	x			
S	PF00018:SH3 domain	X			
S	PF00023:Ankyrin repeat	X			
S	PF00038:Intermediate filament protein	X			
S	PF00069:Protein kinase domain	X			
S	PF00096:Zinc finger, C2H2 type	X	X		
S	PF00169:PH domain			X	
S	PF00439:Bromodomain	X			
S	PF00595:PDZ domain	X			
S	PF00628:PHD-finger	X			
S	PF01388:ARID/BRIGHT DNA binding domain		X		
S	PF01454:MAGE family				X
S	PF02145:Rap/ran-GAP				X
S	PF05110:AF-4 proto-oncoprotein				X
S	PF08447:PAS_3	X			
S	SP_PIR_KEYWORDS:coiled coilt	x			
T	PF00094:VWD				X
T	PF00320:GATA		X		
T	PF00431:CUB domain				X
T	PF00627:UBA/TS-N domain	X			
T	PF01826:Trypsin Inhibitor like cysteine rich domain				X
T	PF02690:Na_Pi_cotrans				X
T	PF08742:C8				X
V	PF00028:Cadherin	X			
V	PF00209:SNF				X
V	PF02263:Guanylate-binding protein, N-terminal domain				X
V	PF02841:GBP_C				X
V	PF08266:Cadherin_2	X			
