# Supplementary material for: Differential Occurrence of Interactions and Interaction Domains in Proteins Containing Homopolymeric Amino Acid Repeats
Source: Front Genet. 2015 Dec 18;6:345. doi: 10.3389/fgene.2015.00345 (PMC4683181; doi:10.3389/fgene.2015.00345)
Supplement: Supplementary Table 3 — Overlap between AARs and protein domains. [file Table3.pdf]

Supplementary table 3 – *Overlap between AARs and protein domains*

AAR	Domain Pfam description	Proteins with AAR + domain in CDD-NCBI	Proteins with AAR + domain in CDD-NCBI with overlap AAR/domain >= 4 residues	%
D	PF01062:Bestrophin	4	4	100.0
I	PF02760:HIN-200/IF120x domain	3	3	100.0
L	PF00503:G-protein alpha subunit	15	15	100.0
R	PF01749:Importin beta binding domain	4	4	100.0
S	PF05110:AF-4 proto-oncoprotein	4	4	100.0
T	PF02690:Na_Pi_cotrans	3	3	100.0
V	PF02263:Guanylate-binding protein, N-terminal domain	7	7	100.0
A	PF00079:Serpin	14	13	92.9
T	PF00320:GATA	5	4	80.0
V	PF00209:SNF	5	4	80.0
F	PF00001:7tm_1	14	11	78.6
R	PF00046:Homeobox domain	22	15	68.2
E	PF06758:DUF1220	16	10	62.5
R	PF00010:Helix-loop-helix DNA-binding domain	16	8	50.0
L	PF00067:Cytochrome P450	25	12	48.0
R	PF01562:Reprolysin family propeptide	8	3	37.5
I	PF00001:7 transmembrane receptor	11	4	36.4
V	PF00028:Cadherin	11	4	36.4
E	PF03160:Calx-beta	6	2	33.3
L	PF00003:7tm_3	9	3	33.3
E	PF00435:Spectrin	10	3	30.0
K	PF00168:C2 domain	16	4	25.0
A	PF00023:Ank	35	6	17.1
L	PF01403:Sema	13	2	15.4
P	PF00041:fn3	27	4	14.8
G	PF00520:Ion transport protein	23	3	13.0
L	PF00005:ABC transporter	16	2	12.5
L	PF00089:Trypsin	34	4	11.8
E	PF00249:Myb-like DNA-binding domain	10	1	10.0
L	PF00092:von Willebrand factor type A domain	21	2	9.5
G	PF02214:K+ channel tetramerisation domain	11	1	9.1
A	PF00076:RBD	40	3	7.5
L	PF00431:CUB domain	15	1	6.7
E	PF00856:SET domain	17	1	5.9
S	PF00096:Zinc finger, C2H2 type	17	1	5.9
L	PF07679:I-set	36	2	5.6
L	PF00028:Cadherin	38	2	5.3
K	PF00628:PHD-finger	25	1	4.0
S	PF00069:Protein kinase domain	56	2	3.6
L	PF00041:Fibronectin type III domain	34	1	2.9
S	PF00169:PH domain	37	1	2.7
