# Supplementary figures and images for: Differential Occurrence of Interactions and Interaction Domains in Proteins Containing Homopolymeric Amino Acid Repeats
Source: Front Genet. 2015 Dec 18;6:345. doi: 10.3389/fgene.2015.00345 (PMC4683181; doi:10.3389/fgene.2015.00345)

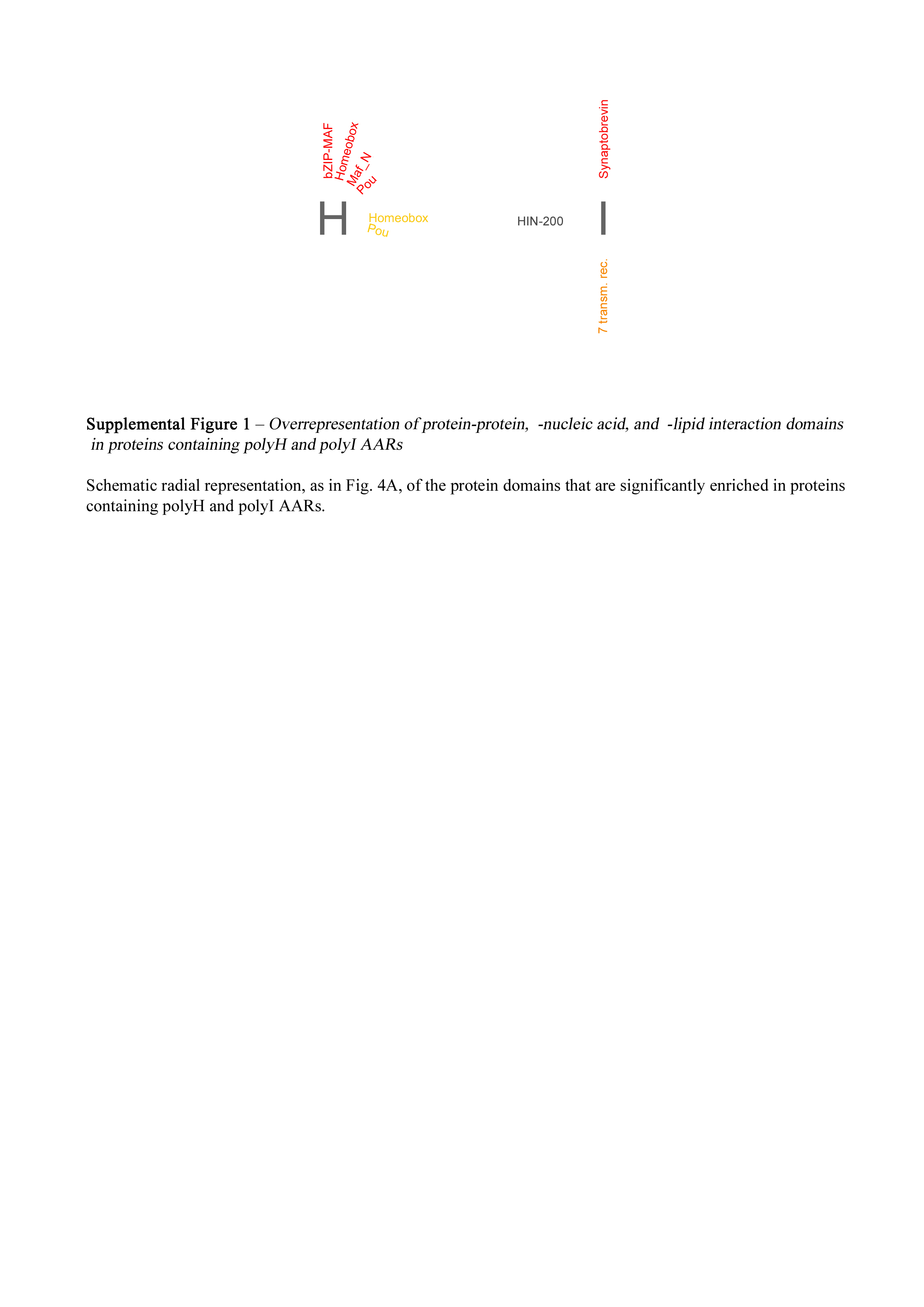

Supplement: Supplementary Figure 1 — Overrepresentation of protein-protein, -nucleic acid, and -lipid interaction domains in proteins containing polyH and polyI AARs. [file Image1.jpg]
